# New Biofuel Alternatives: Integrating Waste Management and Single Cell Oil Production

**DOI:** 10.3390/ijms16059385

**Published:** 2015-04-24

**Authors:** Elia Judith Martínez, Vijaya Raghavan, Fernando González-Andrés, Xiomar Gómez

**Affiliations:** 1 Chemical and Environmental Bioprocess Engineering Department, Natural Resources Institute (IRENA), University of León, Av. de Portugal 41, León 24071, Spain; E-Mails: ejmart@unileon.es (E.J.M.); fernando.gonzalez@unileon.es (F.G.-A.); 2 Department of Bioresource Engineering, McGill University, 21111 Lakeshore Road, Ste-Anne-De-Bellevue, QC H9X-3V9, Canada; E-Mail: vijaya.raghavan@mcgill.ca

**Keywords:** single-cell oil, lipid production, H_2_ fermentative process, organic wastes, lignocellulosic biomass

## Abstract

Concerns about greenhouse gas emissions have increased research efforts into alternatives in bio-based processes. With regard to transport fuel, bioethanol and biodiesel are still the main biofuels used. It is expected that future production of these biofuels will be based on processes using either non-food competing biomasses, or characterised by low CO_2_ emissions. Many microorganisms, such as microalgae, yeast, bacteria and fungi, have the ability to accumulate oils under special culture conditions. Microbial oils might become one of the potential feed-stocks for biodiesel production in the near future. The use of these oils is currently under extensive research in order to reduce production costs associated with the fermentation process, which is a crucial factor to increase economic feasibility. An important way to reduce processing costs is the use of wastes as carbon sources. The aim of the present review is to describe the main aspects related to the use of different oleaginous microorganisms for lipid production and their performance when using bio-wastes. The possibilities for combining hydrogen (H_2_) and lipid production are also explored in an attempt for improving the economic feasibility of the process.

## 1. Introduction

In recent decades, oil prices have been experiencing an increasing trend. Although the present behaviour of the market is resulting in lower oil prices, this may be considered as a temporary situation that may sooner or later come to an end. Concerns about greenhouse gas emissions and high oil prices have promoted research efforts into alternative bio-based processes. However, technologies involving the use of biomass and microorganisms are still far from attaining the goal of transforming the current economic model into a bio-based economy. The main sources used in the energy and transport sector are still petroleum based. However, the gap is closing thanks to the reduction in production costs of bio-based materials. Increments in oil prices also play a primary role in attaining convergence between the traditional economic model and the bio-based model but may also hide a risk of economic slowdown.

Bioethanol and biodiesel are still the main biofuels used in transport applications, with biogas also presenting a few large-scale applications in some localised European areas. The extensive use of liquid biofuels was initially controversial due to the impacts on food prices. Advanced biofuels, although it is a term that is not yet well defined, are rapidly becoming a reality. These fuels may be defined based on the conversion technologies used, which are still in the research and development (R&D), pilot or demonstration phases, and are commonly referred to as second- or third-generation biofuels [1]. In this sense, it is expected that in the near future the production of bioethanol and biodiesel—as well as other advanced biofuels—will be based on processes characterised by low CO_2_ emissions and by the use of organic materials that are different to those traditionally associated with the food-supply chain.

Biodiesel is a non-toxic fuel, making it useful for transport applications in highly sensitive environments, such as marine ecosystems and mining enclosures [2]. The use of biodiesel reduces toxic emissions due to its higher cetane number and lower sulfur content; however, higher NO*x* exhaust emission is a disadvantage [3]. More than 95% of biodiesel production feedstocks come from edible oils, which exert a lot of pressure on the cost of raw materials. Moreover, it is a cause of deforestation in some countries due to the increase in agricultural land required [4,5]. The use of waste cooking oil as a feedstock may represent a reasonable alternative that also solves the problem of waste oil disposal [6,7]. However, the supply is limited by costs associated with collection, transport and pre-treatment, and as a consequence, the price of this raw material is at present close to that of the regular feedstock.

In an attempt to lower the cost and environmental impact of oil-based raw materials, much attention has been paid to the development of microbial oils. Many microorganisms have the ability to accumulate oils under special conditions. Lipids produced by these organisms can be used as potential feedstock for biodiesel production. Compared to plant oils, these oils have several advantages: Short life-cycle, less labour required, less affected by season and climate, and easier to scale up [8,9]. The use of these oils for producing biofuels has been studied only recently [10,11,12]. However, reducing production costs associated with the fermentation process is still of paramount importance to increase economic feasibility, and the use of cheap carbon sources (instead of glucose) is one of the main crucial factors.

The aim of the present review is to describe the main aspects related to the use of different oleaginous microorganisms for lipid production and their performance when using low-cost carbon substrates. A description of the process and improvement of performance by means of combining hydrogen (H_2_) and lipid production is also given.

## 2. Lipid-Accumulating Organisms

Many organisms are able to accumulate lipids under special cultivation conditions. In the case of autotrophic microalgae, carbon dioxide is used as the carbon source while sunlight is the energy source. On the other hand, heterotrophic microalgae can also accumulate oils with organic carbon as the carbon source. Details about lipid accumulation and production by microalgae are out of the scope of this manuscript. The reader is recommended to find information regarding this subject in reviews published by Li *et al.* [10], Gouveia and Oliveira [13] and Sing *et al.* [14].

Lipid-accumulating microorganisms—also called oleaginous microorganisms—are defined as microbes with the capacity to accumulate a lipid content of greater than 20%. Lipids produced from these types of microorganisms are known as single cell oils (SCO) to clearly identify their origin from microbial sources. The oil produced has the same triacylglycerol (TAG) structure as plant oils [15]. As a major component of cell membranes, fatty acids are synthesised in high flux and converted into phospholipids in all organisms. The long hydrocarbon chain is energy-rich, which makes it an ideal precursor for biofuels [16].

Many yeast and mould species accumulate lipids. Fungi have been studied in most cases for producing specific polyunsaturated fatty acids (PUFA). Oleaginous moulds have been extensively studied for the production of high-value PUFA because the oil accumulated by these organisms is characterised by a higher level of unsaturation than lipids accumulated by yeast [17]. Fatty acids—such as ARA (arachidonic acid), DHA (docosahexaenoic acid), GLA (γ-linolenic acid) and EPA (eicosapentaenoic acid)—are of great relevance due to their beneficial effects on the development of the infant brain, eye function and cardiovascular system, among others [18]. Some oleaginous moulds have been used for the production of cocoa butter substitutes [19,20]. However, the availability of cheaper alternatives—as in the case of palm oils and shea nuts—has restrained expansion of this process.

In the case of bacteria, some species have the ability to accumulate oil, but the lipid composition is usually quite different from that of typical yeast strains. Bacteria are not generally lipid-accumulating microorganisms. They usually produce complex lipoids, such as polyhydroxyalkanoic acids, as a means of energy storage and these compounds are deposited as insoluble inclusions in the cytoplasm when a carbon source is available in excess but growth is limited by the lack of another nutrient [21]. However, biosynthesis of TAG seems to be a common feature of bacteria belonging to the actinomycetes group. Members of these genera can accumulate up to 70% of their biomass as lipids. The accumulation takes place mostly during the stationary phase of growth when proteins are not being synthesised and—just as in the case of yeast and mould—these organisms are also highly affected by the type of carbon source and conditions applied. Table 1 presents some experiences of cultivation of lipid-accumulating organisms when using different types of substrates.

**Table 1:** Examples of cultivation of oleaginous microorganisms.

Bacteria	Carbon Sources	Reference
*Rhodococcus opacus* PD630	Carob waste	[22]
*Gordonia* sp. DG	Orange waste	[22]
*Bacillus subtilis*	Glucose	[23]
**Yeast**		
*Lipomyces starkeyi*	Glucose/xylose	[24]
*Cryptococcus curvatus*	Acetic acid Glycerol	[25,26]
*Rhodotorula glutinis*	Distillery wastewater	[27]
*Rhodotorula mucilaginosa*	Hydrolysate of cassava starch	[28]
*Rhodosporidium toruloides*	Glucose	[29]
*Yarrowia lipolytica*	Glucose wastes	[30]
*Trichosporon fermentans*	Glucose	[31]
**Mould**		
*Mortierella isabellina*	Xylose	[32,33]
*Cunninghamella echinulata*	Xylose	[32]
*Thamnidium elegans*	Glucose, fructose and sucrose	[34]
*Mucor* sp.	Cheese whey	[35]

The extent of lipid accumulation is determined by the genetic constitution. The maximum attainable lipid content can vary enormously between species and even between individual strains [36]. Therefore, careful selection of the oleaginous strains of microbial species and characterisation of their lipid composition needs to be performed to ascertain their suitability for biodiesel production [30]. There is also an important dependence between environmental factors, the total amount of lipids accumulated and the type of fatty acid constituents. In this sense, factors such as temperature and substrate concentration may determine the degree of unsaturation [37]. The type of carbon source has a great influence over growth and lipid yields. For example, starch may be an adequate substrate for moulds, but lipids may be produced in lower quantities if compared with yields obtained when using glucose as a sole carbon source [38].

With regard to archaea, over a hundred polar lipids have been identified. These lipids may be used for taxonomic and ecological studies, since they may reflect the phylogenetic relationships of archaeal organisms [39]. The main characteristic of archaeal lipids is the presence of isoprenoids with regularly branching methyl groups bound to glycerol molecules through ether bonds, while in the case of bacteria/eukaryotes, the glycerol moiety is linked to the fatty acids via ester bonds [40]. This distinct configuration makes the direct use of extracted lipids from anaerobic environments impractical for biodiesel production, but opens a new possibility for the production of isoprenoid fuels.

### 2.1. Triacylglycerol (TAG) Biosynthesis in Microorganisms

#### 2.1.1. *De Novo* Lipid Accumulation

In almost all organisms, the biosynthesis of lipids results in the formation of C16 or C18 saturated fatty acids. These fatty acids can be modified through a sequence of desaturases and elongases generating a variety of PUFAs. When sugars are used as the substrate, the process is called “*de novo* lipid accumulation”. In general, oleic acid (∆9C18:1) is the principal fatty acid accumulated with amounts reaching values as high as 70% *w*/*w*, while linoleic (∆9,12C18:2) is found as the second highest [17]. A wide variety of sugars and carbon sources, such as glycerol and cheese whey, have been studied [35,41,42,43].

In order to achieve lipid accumulation, a nutrient limitation needs to be established in the culture medium, with nitrogen usually being the exhausted nutrient. Microorganisms keep assimilating the carbon source, but no further growth is possible due to nitrogen limitation. The carbon flux is then channelled into lipid synthesis, resulting in the accumulation of oil. Not all species are capable of lipid synthesis—in the case of non-oleaginous microorganisms, under similar conditions, cell proliferation simply stops and if carbon assimilation continues to take place, then this is diverted into carbohydrate accumulation, such as glycogen, various glucans and mannans [44].

**Figure 1:**
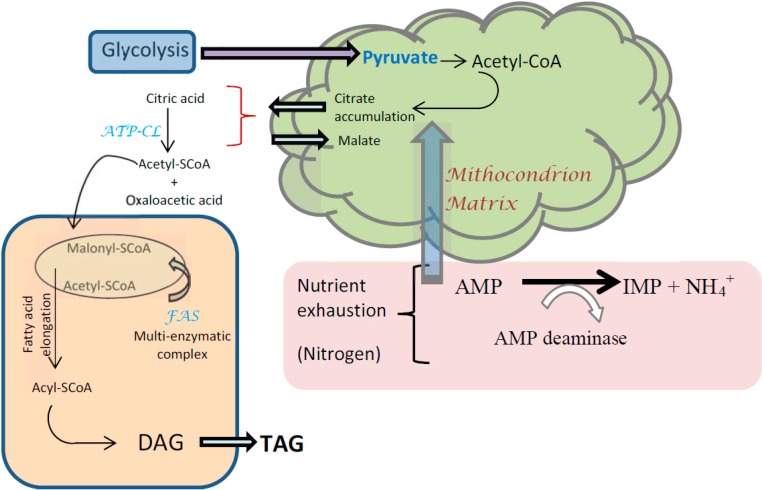
Schematic steps leading to *de novo* lipid biosynthesis and formation of TAG via the α-glycerol phosphate acylation pathway (adapted from [44,45]. IMP: Inosine monophosphate; DAG: Diacylglycerol; TAG: Triacylglycerol).

In oleaginous microorganisms, the fatty acid pathway initiates with the conversion of acetyl-CoA into malonyl-CoA and malonyl-ACP (acyl carrier protein) [16]. Figure 1 represents a brief description of the different stages involved in the formation of TAGs. Acetyl-CoA is transported into the cytosol and derives from the breakdown of citric acid that had previously accumulated inside the mitochondria [17]. The presence of ATP:citrate lyase (ACL) has been identified as being responsible for the formation of acetyl-CoA in oleaginous microorganisms. ACL activity is needed to account for lipid accumulation, but its presence is not a guarantee that the given microorganism is capable of accumulating lipids. In addition, citric acid (which is synthesised as part of the tricarboxylic acid (TCA) cycle) must be available. The unique characteristic associated with lipid accumulating organisms is that the activity of isocitrate dehydrogenase (in the TCA cycle) is dependent on the presence of AMP (adenosine monophosphate), while this dependence does not occur in non-oleaginous species.

AMP deaminase regulates the concentration of AMP. If nitrogen is exhausted, a rapid decrease in intracellular concentration of AMP takes place, and the activity of this enzyme increases as a way of scavenging additional ammonium ions for synthesis of cell material. This results in an alteration in the Krebs cycle with the inhibition of isocitrate dehydrogenase activity and accumulation of citrate until a critical value is reached. Beyond this point, citrate enters the cytoplasm in exchange with malate and is cleaved by ACL to give acetyl-CoA, which is used for fatty acid biosynthesis and oxaloacetate.

Acetyl-CoA carboxylase (ACC) and malonyl-CoA:ACP transacylase are the enzymes responsible for the conversion of acetyl-CoA into malonyl-CoA and malonyl-ACP, respectively [16]. This step is considered as the bottleneck for fatty acid biosynthesis. Acetyl-CoA will generate the cellular fatty acids by a quasi-inverted β-oxidation process. The synthesis of 1 mole of a C18 fatty acid requires 16 moles NADPH. The major supplier of NADPH for fatty acid biosynthesis is considered to be the malic enzyme. However, the activity of this enzyme does not appear to be ubiquitous amongst oleaginous microorganisms and may be absent in some oleaginous yeasts [44]. After the generation of malonyl-CoA, the biosynthesis of fatty acids is performed with the aid of the multi-enzymatic complex of fatty acid synthetase (FAS). The last stage involves the esterification of fatty-CoA ester with glycerol to be stored in the form of TAGs. This synthesis is mainly conducted by the α-glycerol phosphate acylation pathway.

Nitrogen is usually selected as the limiting factor for initiating the storage of lipids. However, phosphorus and sulphate have also been selected as the crucial component for oleaginous yeast, as in the work reported by Wu *et al.* [46,47] for the cultivation of *Rhodosporidium toruloides* under sulphate- and phosphate-limited conditions, obtaining effective lipid accumulation regardless of the presence of high concentrations of nitrogen sources.

A key point for improving lipid production would be the possibility of avoiding aeration of the culture media. This may be achieved by using anaerobic microflora. *Escherichia coli* is widely used as a model system and is close to be converted into a biofuel-production strain. The formation of malonyl-CoA is the first key-limiting step, and therefore the overexpression of ACC, which catalyses the production of malonyl-CoA from acetyl-CoA, in *E. coli* leads to an increase in the rate of fatty acid synthesis [48]. Another approach leading to a significant overproduction of fatty acids is the elimination of feedback inhibition of FAS by hydrolysis of acyl-ACP in combination with the deletion of genes responsible for the β-oxidation pathway. This has made possible a large pool of precursors for the posterior conversion steps into TAGs [49,50]. An engineered *E. coli* cell was developed by Liu *et al.* [51] that was capable of producing 4.5 g/L/day total fatty acid in a fed-batch fermentation system. These authors proved the feasibility of engineering the multi-faceted catalytic and regulatory networks associated with fatty acid biosynthesis. However—from a bioprocessing standpoint—an *E. coli* based process may confront risks associated with bacteriophage attack, which is a serious concern for any industrial fermentation process, but it is a greater challenge when non-aseptic conditions are needed, as would be the case for biofuel production. Bacteriophage (or phage) infect bacteria and cause the fermentation to slow or stop, leading to production downtime and a significant increase in associated costs [52].

Another intensive field of research is the metabolism of *Saccharomyces cerevisiae* as a microbial platform for fatty acid production, fatty acid methyl and ethyl ester (FAMEs and FAEEs) [53]. Enzymes participating in fatty acid biosynthesis in *S. cerevisiae* are encoded by few genes, while in the case of *E. coli*, ten separate genes participate in the process, which makes the overexpression of the entire pathway more straightforward in *S. cerevisiae*. This was the approach presented by Runguphan and Keasling [54] who engineered the budding yeast S. cerevisiae by overexpressing all three primary genes involved in fatty acid biosynthesis, *i.e.*, ACC1, FAS1 and FAS2. The combination of this metabolic engineering strategy with terminal “converting enzymes” (diacylglycerol-acyltransferase, fatty acyl-CoA thioesterase, fatty acyl-CoA reductase, and wax ester synthase for TAG, fatty acid, fatty alcohol and FAEE production, respectively) improved the production levels demonstrating that *S. cerevisiae* provides a compelling platform for a scalable, controllable and economic route for biofuel and chemical production.

#### 2.1.2. *Ex Novo* Lipid Accumulation

The use of fats or hydrophobic materials as the carbon source for lipid accumulation is a growth-coupled process in which the formation of free lipid material takes place in the presence of nitrogen in the culture medium. The fatty material that can be used as a substrate may be free fatty acids, vegetable oils, industrial fats or fish oils [55,56,57]. The degradation of hydrophobic substrates involves the secretion of lipases that present different specificities. The secreted lipases catalyse the hydrolysis of the substrate into free fatty acids, which are later incorporated inside the microbial cell by the aid of active transport or diffuse freely if the concentration gradient is high enough [58,59]. These fatty acids can be used for growth or can be transformed to produce different fatty acids [60]. The different specificities of microbial lipases can lead to different results for the lipid accumulation process. This will affect the fatty acid profile of the stored lipid, which can vary significantly from that of the fat substrate. It can lead either to the storage of more highly unsaturated fat (polyunsaturated fatty acids) or it can lead to dissimilation of the unsaturated form, resulting in the accumulation of more highly saturated cellular lipids than those of the fat substrate.

Fatty acid synthetase and ACL are strongly inhibited by the presence of exogenous *n*-alkanes and fatty acids; therefore, *ex novo* biosynthesis of lipid material cannot take place at the same time as the *de novo* process. However, the yeast *P. methanolica* was reported to accumulate lipid material in the presence of glucose and a triacylglycerol rich in docosahexaenoic acid (DHA), possibly because *P. methanolica* incorporated lipids from the medium without hydrolysis, at least in part, and accumulated them without any transformation in the cells [61].

On the other hand, free fatty acids are converted into acyl-CoA esters by acyl-CoA synthetases (ACS) and degraded into smaller chain acyl-CoA ester and acetyl-CoA by the β-oxidation process. These reactions are catalysed by various acyl-CoA oxidases (Aox), therefore providing the energy necessary for cell growth, maintenance and the production of intermediate metabolites. The shortening of the chain of acyl-CoA ester is a process consisting of a four-reaction cycle that results in the loss of two carbons (acetyl-CoA). This process is repeated several times until the complete degradation of the fatty acid is attained, but it can be stopped earlier depending on the length and concentration of the substrate, the presence of acetyl-CoA and on the ratio of NAD^+^/NADH [59]. Depending on the operating conditions of the cultures, yeast cells can degrade fatty acids or incorporate them as TAG and steryl esters (STE) into lipid bodies. The structure of this organelle consists of a lipid core encased by a phospholipid monolayer embedded with numerous proteins [62]. The main interest in exploring *ex novo* lipid accumulation is associated with the modification of the hydrophobic substrate to produce high-value PUFAs.

## 3. Reducing Fermentation Costs with the Use of Wastes and Lignocellulosic Biomass

The use of low-cost carbon sources for the production of SCO has been extensively studied as a means of reducing costs, and thus becoming competitive with traditional energy crops for oil production [5,8,22]. The fermentation process must cope with seasonal and local availability of the carbon source, and the cost of transport of the substrate and product distribution. The hypothetical use of SCO makes it necessary to be able to obtain cheap raw materials all year round, to increase fermentation yields by genetic manipulation of specific strains and to improve bioreactor performances, particularly under continuous operation. The economic feasibility of the lipid production process is not only determined by the cost of raw materials, but also by the operating conditions of the fermentation, which will affect the volumetric production of the fermenter [63].

In bulk-scale production, the environment is typically not sterile and microbial biomass may be reused in consecutive batches. The low growth rates of manipulated strains may become a problem for industrial applications. If sterilisation needs to be avoided, these strains will not be able to compete against wild-type species in a non-sterile environment [64]. Therefore, the evaluation of performance under non-sterile conditions is of great relevance. Santamauro *et al.* [65] studied the yeast *Metschnikowia pulcherrima* for its potential to produce lipids for biofuel production. The yeast was cultured in a glycerol media in two 500-L raceway ponds at 21 °C. This approach resulted in yields of up to 40% lipid content, which compares favourably with other oleaginous microbes.

The use of wastes as substrates for the production of SCO for biofuels has recently been studied. Substrates, such as glycerol, food-processing wastes, and cheese whey, have already been evaluated due to their high sugar content. Molasses has not been traditionally studied as a suitable substrate due to its high content of organic nitrogen [66], which can constitute approximately 8%–12% (*w*/*w*) in sugar beet molasses [67]. Optimum C:N conditions are crucial for allowing cells to initiate lipid storage. Lipid accumulation is usually optimal at a molar C:N ratio greater than 65 and near 100 [68] with temperatures around 20–28 °C and pH 4–7 for yeasts and moulds [17,63]. However, good results were reported by Chatzifragkou *et al.* [43] when studying the growth of *Cunninghamella echinulata* and *Mortierella isabellina* fungi using sugarcane molasses, which is characterised by low nitrogen content of 28%–43% (*w*/*w*) [69]. One point to be addressed is the indirect social pressure exerted while using low cost substrates in many industrial activities viz., cheese whey, molasses and food-industry wastes are still categorised as animal feed in many countries. Diverting their use to other industrial processes may affect the market price of human consumption products.

Glycerol and crude glycerol have been extensively studied as substrates for lipid production. Lipid contents reported in the literature are 21%–35% (*w*/*w*) for *Rhodotorula glutinis* [70,71], and 43%–53% (*w*/*w*) for *Cryptococcus curvatus* [41,72]. The conversion of crude glycerol is an interesting option for increasing the productivity of biodiesel plants, which need to cope with high prices of raw materials. Table 2 presents the results of lipid yields obtained by different authors.

**Table 2:** Lipid yields reported in the literature from different wastes and lignocellulosic biomass.

Substrate	Microorganism	Lipid Yields (g Lipid/g Biomass)	Reference
**High carbohydrate content**			
Glucose derived from maize starch hydrolysate	*Mortierella alpina*	0.33–0.36	[73]
Molasses	*Candida lipolytica*, *Candida tropicalis*, *Rhodotorula mucilaginosa*	0.16–0.60	[74]
		0.12–0.46	
		0.39–0.69	
Glycerol	*Mortierella alpina*	0.05–0.33	[42]
Crude glycerol	*Cryptococcus curvatus*	0.44–0.52	[41]
**Lignocellulosic material**			
Rice hull hydrolysate	*Mortierella isabellina*	0.64	[75]
Cassava starch hydrolysate	*Rhodosporidium toruloides*	0.63	[76]
Corncobs	*Trichosporon dermatis*	0.17	[77]
Corn stover	*Cryptococcus curvatus*	0.16	[78]
Rice straw hydrolysate	*Trichosporon fermentans*	0.4	[79]
Wheat straw	*Cryptococcus curvatus*	0.05	[80]
**Complex substrates**			
Distillery wastewater	*Rhodotorula glutinis*, *Cryptococcus curvatus*	0.25	[27]
		0.27	
Pre–treated sewage sludge (ultrasounds)	*Lipomyces starkeyi*	0.32–0.35	[81]
Waste cooking oil	*Yarrowia lipolytica*	0.17–0.55	[30]
Waste motor oil			
Palm oil mill effluent	*Rhodotorula glutinis*	0.21–0.38	[82]

The use of low-cost greases may be a suitable alternative for the valorisation of these substrates. Used cooking oil (UCO) is a cheap feedstock that is widely used for biodiesel production due to its lower price than that of refined vegetable oils. In addition, the valorisation of this oil prevents environmental contamination if no proper disposal method is implemented [7,83]. This substrate may be directly used for lipid-accumulating organisms. *Yarrowia lipolytica* has a special ability for the use of fats and oils. There are several studies reporting the conversion of hydrophobic substrates by this organism, but most of them are intended for the production of high-value fats like cocoa [84] and PUFAs [85], using also in this latter case, genetically modified strains [17]. The study of Katre *et al.* [30] is one of the few reports of the conversion of waste cooking oil and motor oil into valuable lipids.

Lignocellulosic material, on the other hand, is a suitable substrate for producing biofuels due to its high availability and low risk of affecting food prices. In addition, the production of agricultural biomass and its exploitation for energy purposes can contribute to alleviating the dependence on fossil fuels and offers a unique opportunity for potentiating the agricultural sector and reducing the abandonment of land by farmers [86]. Lignocellulose comprises an important fraction of municipal solid wastes, energy crops and forest residues [87]. Different lignocellulosic and organic wastes have been tested as carbon sources for the production of SCO [22,78,80]. However, production of biofuels from cellulosic biomass generally requires the previous conversion of cellulosic material into simple sugars, and subsequent conversion of these sugars into biofuels. The pre-treatment needed for this step is usually energy intensive and may require using either hazardous chemicals or a large amount of enzymes during the hydrolysis step, which can significantly increase costs and therefore limit its industrial applications. Physical, chemical and enzymatic pre-treatments have been proposed for the conversion of lignocellulosic biomass. The pre-treatment is required to reduce the recalcitrance of lignocellulosic material by opening or partially breaking the recalcitrant structure, while minimising the chemical degradation of fermentable sugars [88]. Among the different pre-treatment methods, the ethanol organosolv process is the most promising, since it results in a clean fractionation of biomass into a cellulose-rich residue, which is further digested by cellulase giving high glucose conversion [89].

There exists vast experience from the ethanol industry with several demonstration plants being installed for the use of lignocellulosic biomass as the substrate for bioethanol production. A lot of effort has gone into the development of processes that biologically convert lignocellulosic biomass into simple sugars (C5 and C6), but in many cases, there is still a need to apply a mild chemical or physical pre-treatment to increase digestability of the encapsulated cellulose [90]. The quality of sugars obtained from the conversion of lignocellulosic biomass depends on the physico-chemical characteristics of the raw material, and also on the type of pre-treatment selected. Harsh conditions may lead to the production of possible toxic compounds due to the partial degradation of hemicellulose [91]. The presence of toxic compounds, such as furfural, is known to negatively affect the fermentation process. This toxic compound has been reported to inhibit cell growth and lipid content by 72.0% and 62.0%, respectively, at a concentration of 1 g/L when tested on *C. curvatus* growth using wheat straw hydrolysates [80]. It is of particular importance to gain a better understanding of the inhibition mechanisms and the negative effects on lipid accumulation in order to obtain solutions for its minimisation and to be able to develop processes operating at high loading conditions [45]. *Y. lipolytica* is a well-known oleaginous yeast that has been extensively investigated to explore its potential for biodiesel production when grown on inexpensive wastes. This oleaginous yeast produces SCO (58.5%) using sugarcane bagasse hydrolysate as substrate [92], but poor performance was observed when grown on wheat straw hydrolysate [80].

In addition, compatibility of the fermentation is of considerable relevance since xylose is the second-most abundant sugar generated from the pre-treatment of lignocelluloses. Therefore, the selection of an organism capable of fermenting C5 and C6 sugars may directly result in higher efficiencies for the global process. In the case of glucose metabolism, about 1.1 moles of acetyl-CoA are generated from 100 g of glucose catabolised, but in the case of xylose, and considering the same mass base, the yield would be either 1.2 or 1.0 mole if it is metabolised through the phosphoketolase reaction in the first case or the pentose phosphate pathway in the second case. Based on these assumptions, the maximum theoretical yield of SCO produced would be around 0.32 g/g-based on a substrate basis-when considering glucose as the substrate and around 0.34 g/g when xylose is the substrate [32,93] (assuming as previous condition that all acetyl-CoA produced was channelled towards the synthesis of lipids). However, lipid yields on glucose are in practice lower than 0.2 g/g [32,94].

Fermenting with the use of *Candida tropicalis* is of particular interest because this yeast strain is capable of catabolising glucose and xylose as carbon sources. When using glucose as the substrate, the reported lipid yield was 0.176 g/g at a nitrogen-stress molar ratio of 150:1 (C:N) [95]. Even though the co-fermentation of glucose and xylose using pure cultures may be a guarantee of high lipid yields, the need of substrate and equipment sterilisation for industrial applications may become a burden. An interesting option has been proposed by Mondala *et al.* of using waste activated sludge as microbial cultures. Fermentation yields reported by these authors were much lower (0.02 to 0.06 g/g) when compared with pure cultures, but the co-fermentation of glucose:xylose mixtures at 2:1 and 1:2 (by mass) was successfully obtained [96]. Use of sugarcane bagasse hydrolysate as the substrate resulted in lipid contents from 40% to 47% (dry cell weight) under a high C:N ratio (70:1) [97]. These results demonstrate potential for the future of non-sterile process intended for waste valorisation.

A novel alternative to increase the global yield of the fermentation process involves the use of lignin, for example, using oleaginous *Rhodococci* with lignin as the carbon source. Lignin is generally regarded as a by-product from the pre-treatment process of lignocellulosic biomass. In the case of thermal pre-treatment, lignin is usually valorised by means of combustion for heat recovery. Some bacteria, such as *Rhodococcus opacus* DSM 1069, can degrade this compound and accumulate lipids up to 4.08% [98,99]. Even though this value may seem low, it is the first step for an innovative application in the use of lignin as a carbon source, since the degradation of this complex compound has been exclusively assigned to fungi species in the past.

## 4. Improving Lipid Production from Wastes

Increasing productivity of lipid-producing organisms should be the main objective in studies for attaining process profitability. Even though data reported in the literature present high values of lipid accumulation in cell biomass (as expressed in % *w*/*w*), these same values when expressed in volumetric production may seem quite low. For example, Liang *et al.* [41] reported a lipid productivity of 1.5 g/L day when using crude glycerol as the substrate. Increasing lipid concentration may be attained by an increase in the concentration of substrates fed into the reactor and by operating under a fed-batch configuration [100]. High-density cultivation using oleaginous yeast *Rhorosporidium toruloides* Y4 was studied with glucose as the carbon source by Li *et al.* [29]. Experiments were carried out in a pilot-scale fed-batch fermenter (15-L stirred-tank reactor) obtaining a biomass concentration of 106.5 g/L and lipid productivity of about 13 g/L day without observing significant inhibitory effects at a substrate concentration of up to 150 g/L.

The use of lignocellulosic material, which is characterised by high solid content, may point to a new direction in fermentation trends. The use of sweet sorghum at high solid concentrations by *Rhodosporidium toruloides* was demonstrated by Matsakas *et al.* [101]; the addition of enzymes permitted liquid fermentation at high substrate concentration and enhanced lipid production by 85.1% and 15.9% when dried stalks or stalk sorghum juice were used, respectively. SCO production was 13.77 g/L, which is one of the highest titres reported in the literature when using low-cost substrates.

On the other hand, solid-state fermentation (SSF) may be a powerful platform for the effective transformation of agro-materials. The key to success for this fermentation configuration is the selections of fungal strains with high efficiency to utilise low-priced substrates and reduce posterior processing [102]. The main advantage of SSF is the absence of a free aqueous phase. This results in minimum water consumption and the possibility of conducting the processes under semi-sterile conditions since the growth of contaminating bacteria is minimised [103]. This is an interesting feature for the industrial use of agricultural wastes as carbon and energy sources for biofuel production. However, the need for pre-treatment may limit the application of this technology unless a combination of saccharification and fermentation could be attained. In this latter case, optimum operating conditions for the enzymatic hydrolysis process and simultaneous fermentation need to be found which is not an easy task.

The vast experience gained in the production of ethanol is useful for adapting operating requirements for SCO production. The initial saccharification stage is usually performed at higher temperatures than would be inadequate for the growth of microbial oil producers, while differences in optimum pH values are smaller. It should be borne in mind that this simultaneous process still needs an initial pre-treatment of the lignocellulosic biomass to increase the accessible surface area to favour enzymatic degradation. Several authors have reported the use of cellulases for performing the combined process for ethanol production. However, the operation temperature is recommended to be around 35–37 °C, otherwise the low hydrolysis rate would result in a poor fermentation performance [104,105]. These temperatures are still much higher than those usually recommended for SCO-producing microbes, and therefore are an important constraint to the combined process. An alternative may be to explore the feasibility of culturing thermophilic organisms. Zheng *et al.* [106] cultured *Thermomyces lanuginosus* using xylose and wheat straw hydrolysate as the carbon source and reported successful performance at 50 °C. One additional advantage is the fact that, in contrast to ethanol fermentation, acetic acid can also be used as a substrate for lipid-producing microorganisms. This acid is typically produced during pre-treatment of lignocellulosic biomass due to de-acetylation of hemicellulose, and it is considered as an inhibitor in the ethanol production process, but as a substrate when producing lipids.

The use of acetic acid may become the linking point for combining hydrogen (H_2_) and lipid production; the combination of these two processes has been reported by Bagy *et al.* [107] for the fermentation of the spent liquor of fungal cultures on sugarcane molasses. In this case, the authors tested the use of residual sugars by *Clostridium acetobutylicum*. However, the fermentative H_2_ process is characterised by low productivity since only 33% of the chemical oxygen demand (COD) can be transformed into H_2_ (considering glucose) with the remainder being mainly composed of volatile fatty acids (VFAs, e.g., acetate, butyrate) [108,109]. Taking into consideration the low efficiency of the H_2_ fermentation process, the intuitive approach should be to perform an initial stage for H_2_ production, and a subsequent step for microbial lipid production. In fact, this is the configuration studied by Chi and co-workers [110]. VFAs from fermentative hydrogen production were tested as carbon sources for the culture of oleaginous yeast *Cryptococcus curvatus*. This organism could be easily adapted to conditions encountered in the acidic food-waste effluent from the H_2_ fermentation stage. However, the lipid content (in biomass) reported by the authors was low due to the high protein content in food waste. This result suggests that raw materials with low nitrogen content, such as lignocellulosics, may be adequately treated in a two-phase configuration for H_2_ and lipid production.

Another additional advantage offered by the H_2_ fermentative process is the availability of degrading cellulosic material, which is possible with the use of thermophilic microorganisms. *Clostridium thermocellum* is an anaerobic bacterium that is capable of metabolising cellulose with the concomitant release of H_2_ [111]. Successful production of H_2_ from cellulose has also been reported by Geng *et al.* [112], but their study indicated the need for addition of an alkali solution to increase the assimilation of cellulose to a value of 94.3%. Similarly, Cao *et al.* [113] reported successful results (155 mL H_2_/g VS, volatile solid) with the use of alkali pre-treated cornstalks using mixed-microflora incubated at 60 °C for H_2_ production. The direct use of lignocellulosic material for the production of H_2_ has been demonstrated by Magnusson *et al.* using barley hulls [114], and Chen *et al.* using rice straw [115]. New strains of thermophilic cellulolytic bacteria designated as *Thermoanaerobacterium thermosaccharolyticum* M18 have been isolated by Cao and colleagues [116]. These authors reported the use of natural lignocellulosic materials without any physicochemical or biological pre-treatment for the growth of strain M18. H_2_ yields when using corncob, cornstalk, and wheat straw as substrate were 72.4–77.9 mL H_2_/g substrate.

The results described above indicate that the two-stage configuration for H_2_ and lipid production opens up new possibilities for integrating the valorisation of agro-industrial and organic wastes. There is a need for establishing operating conditions for the combined process and to identify possible inhibitory compounds and their effect on both microbial cultures, but the combination of these two processes may become an important alternative for reducing operating costs in biofuel production processes.

## 5. Conclusions

The production of SCO from organic and lignocellulosic waste has undergone extensive research in an attempt to increase the lipid productivity and to reduce fermentation costs. The process offers new possibilities for the valorisation of wastes. However, the increase in microbial concentration should be attained in order to increase lipid daily productivity. In this sense, fermentation experiences have been evaluated operating under fed-batch configuration and high substrate concentration. Considering the particularities of lignocellulosic wastes and many other organic wastes, operating under a solid-state configuration could be a suitable alternative. On the other hand, the combined process for H_2_ and lipid production may become an important future line of research. The fermentative H_2_ production process is characterised by the concomitant conversion of the organic matter into H_2_ and short-chain organic acids that could serve as a substrate for a secondary fermentation intended for lipid accumulation. The use of thermophilic temperatures may allow the direct use of lignocellulosic substrate and avoid the need of energy-intensive pre-treatment operations.

Engineered organisms using *E. coli* and *S. cerevisiae* platforms are an interesting option, but are still full of challenges if industrial application and large-scale production are intended. Biofuels are a commodity that needs to be competitive with the oil industry products, and therefore, the operating cost is a major concern. A bio-based economy using engineered microorganisms may be promising, but multiples aspects need to be dealt with before this option can become a reality. Sterile condition, pre-treatments, raw material costs and contamination by different microflora are aspects still needing a solution.
